# Underwater endoscopic submucosal dissection via continuous irrigation method and red dichromatic imaging for a rectal cancer with severe fibrosis

**DOI:** 10.1055/a-2709-7410

**Published:** 2025-10-07

**Authors:** Takahiro Muramatsu, Masakatsu Fukuzawa, Fumito Yamanishi, Makoto Arashiyama, Fumi Naruse, Ai Enomoto, Takao Itoi

**Affiliations:** 1 38548 Department of Gastroenterology and Hepatology, Tokyo Medical University Hospital, Tokyo, Japan; 2 38548 Department of Anatomic Pathology, Tokyo Medical University Hospital, Tokyo, Japan

Endoscopic submucosal dissection (ESD) for colorectal tumors with fibrosis is technically challenging due to the risks of perforation and difficulty in achieving en bloc resection. Submucosal invasive cancer is known to be a risk factor for severe fibrosis 1 2 . We previously reported using red dichromatic imaging (RDI) 3 4 and the continuous irrigation method (CIM) 5 to facilitate difficult resections. Herein, we report a case of successful en bloc resection of a submucosal invasive cancer with fibrosis in the lower rectum using RDI and CIM ( Video 1 ).

Underwater endoscopic submucosal dissection via continuous irrigation method and red dichromatic imaging for a rectal cancer with severe fibrosis.Video 1

An 86-year-old woman presented with a 25-mm 0-Is lesion in the lower rectum. Endoscopic findings suggested deep submucosal invasion ( Fig. 1 ). Although surgery was recommended, the patient declined due to her advanced age, and ESD was performed.

**Fig. 1:**
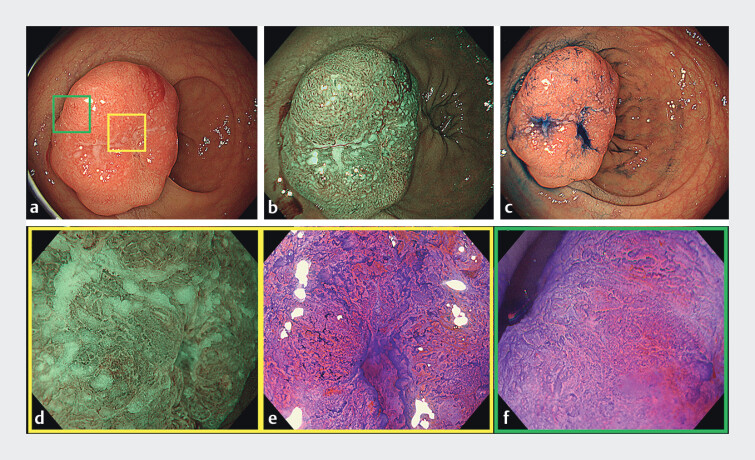
Endoscopic images. **a** White-light image. Lower gastrointestinal endoscopy revealed a protruding lesion (25 mm, type 0-Is) in the lower rectum. **b** Narrow-band imaging (NBI) view. **c** Indigo carmine dyeing view. The depression in the center of the lesion was a biopsy scar from a previous physician. **d** Magnified NBI view of the yellow square in Fig. 1 **a** . Irregular microvessels and surface patterns were observed, corresponding to Japan NBI Expert Classification type 2B. **e** Magnified endoscopy with crystal violet staining of the yellow square in Fig. 1 **a** revealed a V _I_ high-grade pit pattern. **f** Magnified endoscopy with crystal violet staining of the green square in Fig. 1 **a** also revealed a V _I_ high-grade pit pattern.

Underwater ESD was initiated; however, the endoscopic view was impaired by bleeding and bubbles. Therefore, CIM was deployed, effectively clearing the endoscopic view ( Fig. 2 **a–d** ). Circumferential mucosal incision was completed under CIM ( Fig. 2 **e** ). RDI improved visualization of the boundary between the submucosal and muscular layers, while the water pressure effect from CIM supported submucosal dissection and provided tamponade hemostasis during bleeding ( Fig. 2 **f–i** , Fig. 3 **a–g** ). Even in fibrotic areas, the combination of CIM and RDI enabled clear identification of the dissection plane, resulting in en bloc resection without adverse events ( Fig. 2 **j–l** , Fig. 3 **h–l** ).

**Fig. 2:**
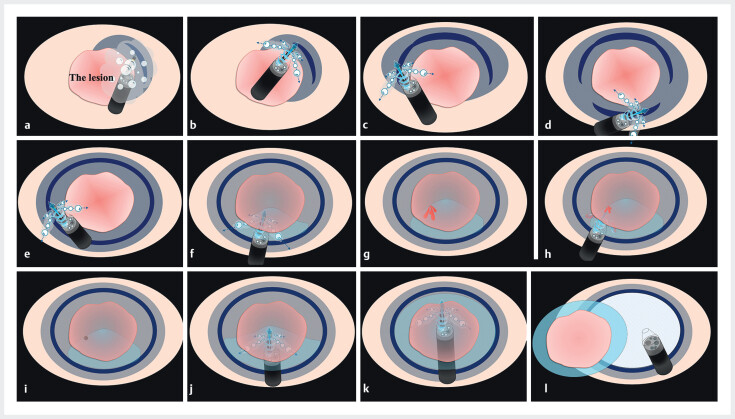
Schematic diagram illustrating the steps of lesion resection with underwater endoscopic submucosal dissection using the continuous irrigation method (CIM). **a** A mucosal incision was made on the distal side of the lesion. Bleeding and air bubbles associated with the mucosal incision impaired the endoscopic view. **b** By employing CIM, blood and bubbles were efficiently displaced, resulting in a consistently clear endoscopic field. **c** The mucosal incision was continued using CIM. **d** A mucosal incision was also made on the proximal side of the lesion using CIM. **e** A whole circumferential incision was made. **f** Submucosal dissection was performed using CIM. **g** Bleeding occurred. **h** By using CIM, bleeding and bubbles were cleared, resulting in an improved endoscopic view. In addition, the tamponade effect of CIM facilitated the identification of the bleeding point. **i** Complete hemostasis was supported by CIM. **j** Submucosal dissection for the fibrotic area was also performed using CIM. **k** Submucosal dissection continued with CIM. **l** Complete en bloc resection was achieved.

**Fig. 3:**
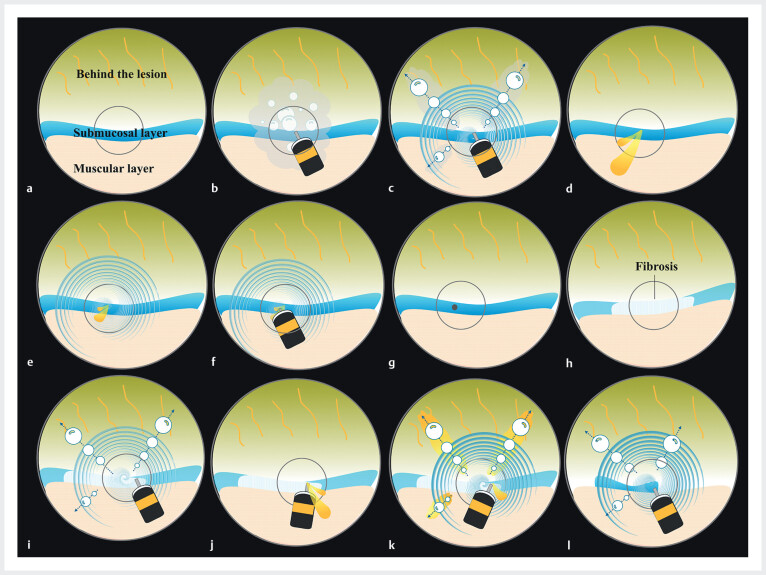
Schematic illustration of the endoscopic view under red dichromatic imaging. **a** The view upon entering the submucosal layer. **b** Bleeding and bubbles associated with the dissection resulted in a poor endoscopic view. **c** After deploying the continuous irrigation method (CIM), bleeding and bubbles were blown out, and the field of view became clear. **d** Bleeding occurred. **e** The water pressure of CIM weakened the force of bleeding. **f** CIM provided a temporary tamponade effect, supporting hemostasis. **g** Complete hemostasis was achieved by CIM. **h** Approaching the fibrotic area. **i** Even in fibrotic areas, CIM blew away bubbles and contributed to identifying the dissection line under a clear endoscopic view. **j** Minor bleeding occurred. **k** In cases of minor bleeding, CIM could blow away the blood, allowing dissection to proceed with simultaneous hemostasis. **l** After passing through the fibrotic area, the remaining submucosal layer was dissected using CIM.

Histopathological analysis revealed a deep submucosal invasive cancer with lymphovascular invasion and grade 3 budding ( Fig. 4 ). The patient later underwent additional surgery, which confirmed curative resection.

**Fig. 4:**
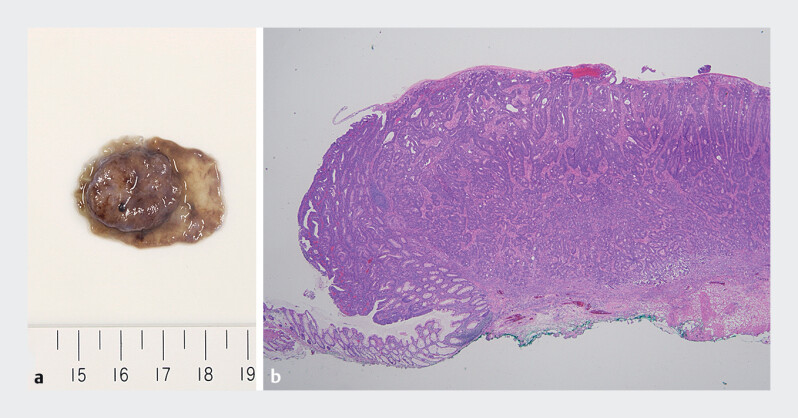
Macroscopic and histopathological images of the resected specimen. **a** Macroscopic view of the specimen. **b** Histopathological image of the specimen. The pathological diagnosis revealed T1 cancer with deep submucosal invasion (5000 µm) with positive lymphovascular invasion, and grade 3 budding. The horizontal margin was negative, but the vertical margin was inconclusive.

CIM offers a simple setting that improves poor visibility due to bleeding or bubbles and allows for the smooth continuation of the procedure. RDI further enhances visualization of fibrotic and submucosal structures. The combination of CIM and RDI may provide a safe and effective method for resecting fibrotic colorectal lesions.

Endoscopy_UCTN_Code_TTT_1AQ_2AD_3AD

## Notes

Endoscopy E-Videos https://eref.thieme.de/e-videos
*E-Videos* is an open access online section of the journal *Endoscopy* , reporting on interesting cases and new techniques in gastroenterological endoscopy. All papers include a high-quality video and are published with a Creative Commons CC-BY license. Endoscopy E-Videos qualify for HINARI discounts and waivers and eligibility is automatically checked during the submission process. We grant 100% waivers to articles whose corresponding authors are based in Group A countries and 50% waivers to those who are based in Group B countries as classified by Research4Life (see: https://www.research4life.org/access/eligibility/ ). This section has its own submission website at https://mc.manuscriptcentral.com/e-videos .
